# Tritium Release and Mechanical Properties of Advanced Tritium Breeder: Li_4_Si_0.8_Ti_0.2_O_4_ Ceramic Pebbles

**DOI:** 10.3390/ma19122536

**Published:** 2026-06-11

**Authors:** Juemin Yan, Nanlin He, Baoping Gong, Hao Cheng, Long Zhang, Xiaoyu Wang

**Affiliations:** 1 Southwestern Institute of Physics, Chengdu 610041, China; yanjuemin@swip.ac.cn (J.Y.); gongbp@swip.ac.cn (B.G.); chenghao@swip.ac.cn (H.C.); 2 Basic Department, Police Officer College of Armed Police Force, Chengdu 610065, China; hnlemail@126.com

**Keywords:** tritium breeder materials, Li_4_Si_0.8_Ti_0.2_O_4_, irradiated with different neutron, tritium release, mechanical properties

## Abstract

Lithium-containing ceramics were significant tritium breeders for the fusion blanket concept, for which tritium release performance and mechanical properties serve as the core indicators for evaluating their performance as tritium breeders. The Li_4_Si_0.8_Ti_0.2_O_4_ material was designed as an advanced tritium breeder and fabricated into ceramic pebbles via the freeze-drying method. The tritium release properties of the Li_4_Si_0.8_Ti_0.2_O_4_ sample pebbles were investigated via temperature-programmed desorption (TPD). The mechanical properties of the same batch of tritium breeder pebbles were analyzed comparatively, specifically examining the change in their compressive strength before and after irradiation. The sample pebbles irradiated with different neutron doses show different tritium release characteristics, and the tritium release temperature was about 293–553 °C. This was due to the H_2_-tritium isotope exchange reaction, and radiation with different neutron doses will lead to different release temperatures of tritium. The mechanical properties of the Li_4_Si_0.8_Ti_0.2_O_4_ ceramic pebbles decreased significantly after irradiation. The main reason was that the accumulation of lattice defects and helium bubbles produced by high-energy neutron irradiation leads to internal cracks and helium embrittlement in the material. These results indicate that Li_4_Si_0.8_Ti_0.2_O_4_ solid solution may be considered a potential candidate for tritium breeder materials.

## 1. Introduction

The solid-state tritium breeding blanket was important core component of fusion reactor, primarily responsible for the functions of tritium breeding and energy conversion [1]. Tritium generation was mainly achieved through neutron bombardment of lithium materials, and tritium itself is a radioactive isotope of hydrogen. This lithium-containing material is referred to as tritium breeder materials [2]. Tritium breeder materials play a crucial role because they constitute a major component of the fusion blanket. The primary function of a breeder material is to ensure tritium self-sufficiency via the lithium-6 neutron interaction [3,4]. Lithium-based ceramics, which possess excellent physical properties and chemical durability, are the most promising tritium breeder materials for tritium generation [4,5,6,7,8,9,10,11,12]. A desired breeder material should possess high lithium density, advanced mechanical properties, good thermal conductivity, low activation performance, and efficient tritium release. Lithium orthosilicate (Li_4_SiO_4_) is a highly significant tritium breeder candidate for China’s test blanket model (TBM) [13,14,15].

Although Li_4_SiO_4_ exhibits relatively favorable tritium release performance at lower temperatures, its mechanical properties are generally unsatisfactory. It has been demonstrated that ion diffusion in Li_4_SiO_4_-based ceramic materials can be substantially enhanced by substituting the Si or Li in them with B^3+^, P^5+^, Ti^4+^, V^5+^, Co^2+^, Ni^2+^, or Ga^3+^ (different cations), which may increase the strength [16]. While extensive in-pile and out-of-pile experiments have examined the effects of parameters such as material properties, purge gas chemistry, and irradiation defects on tritium release behaviors in ceramic breeder materials [17,18], further efforts have been devoted to understanding the relationship between the diffusion characteristics of tritium and the diffusion ability of lithium ions in solid tritium breeding materials [19,20]. The doping elements may facilitate the total tritium recovery and boost the ion migration rate. Meanwhile, to improve the mechanical properties of Li_4_SiO_4_ and explore the tritium release property of doped-Li_4_SiO_4_ ceramic materials, we used the Ti element as the replacement because it has the same charge and large atomic radius as the Si element. As the Ti-O bond was stronger than the Si-O bond, the Li-O bond reciprocity was reduced in the Li_4_SiO_4_ structure when Si was replaced by Ti.

Numerous techniques have been developed for the fabrication of ceramic breeder pebbles, such as melting–spraying [21], extrusion–spheronisation–sintering [22], agglomeration–sintering [23], sol-gel [24], and wet processes [12]. Nevertheless, these techniques exhibit different advantages in the fabrication of ceramic pebbles. In the present work, a wet process was used in the fabrication process of Ti-doped Li_4_SiO_4_ (Li_4_Si_0.8_Ti_0.2_O_4_) ceramic pebbles take advantage of its economy and cleanliness. The tritium release behavior of breeder materials is of critical importance for the design of the tritium fuel cycle in fusion energy conversion. Therefore, the investigation of tritium release properties from Li_4_Si_0.8_Ti_0.2_O_4_ ceramic pebbles was inevitable.

## 2. Experimental Methodology

### 2.1. Fabrication of Sample Pebbles

The fabrication of polycrystalline Li_4_Si_0.8_Ti_0.2_O_4_ pebbles can be accomplished in two-step process: the synthesis of powder samples and the preparation of pebbles. Firstly, Li_4_Si_0.8_Ti_0.2_O_4_ powder samples were synthesized using the solid-state reaction. LiOH·H_2_O (Aladdin Chemistry Co. Ltd., Shanghai, China, 99.0%), SiO_2_ (Shanghai Kefeng Chemicals, Shanghai, China, 99.99%), and TiO_2_ (Alfa Aesar Chemicals Co. Ltd., Shanghai, China, 99.8%) were selected as starting material. First, the raw materials were mixed stoichiometrically in aqueous solution and heated at 900 °C for 10 h. The Li_4_Si_0.8_Ti_0.2_O_4_ powder were synthesized as follows:

(1) $$4LiOH\cdot H_{2}O+0.8SiO_{2}+0.2TiO_{2}\to {Li}_{4}{Si}_{0.8}{Ti}_{0.2}O_{4}+5H_{2}O$$

**Figure 1:**
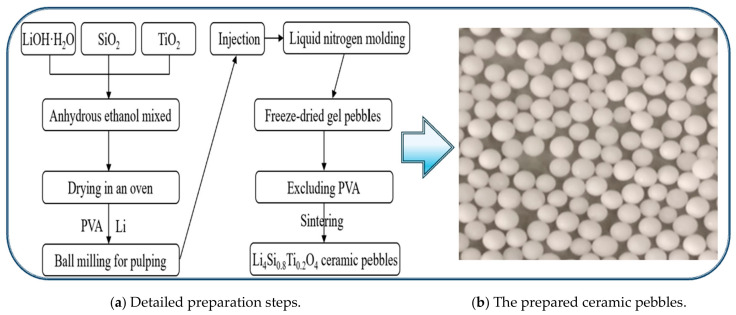
The detailed process for preparing Li_4_Si_0.8_Ti_0.2_O_4_ ceramic pebbles.

The second step involved preparing sample spheres using a wet process. Using 50 g of Li_4_Si_0.8_Ti_0.2_O_4_ powder (with a Si/Ti molar ratio of 4) as the raw material, 3% aqueous polyvinyl alcohol solution was added at a solid–liquid mass ratio of 1:1.5. At this point, the total mass of the slurry was approximately 125 g, with a solid content of 41.8 wt.% and a liquid content of 58.2 wt.%. The above mixture was then placed in a ball mill and ball-milled to form a stable slurry. After ball milling, a stable slurry was formed. Then, the slurry was dropped into the liquid nitrogen through the injection nozzle to generate gel pebbles. In this step, the formation of sample pebbles was from surface tension function, and the drippage of pebbles was powered by gravity action. The main function of liquid nitrogen was to hold the sphere’s shape. The spheres were freeze-dried under high vacuum, and the temperature was maintained below ice point in quick succession. Next, the pebbles were sintered at 650 °C for 6 h (remove polyvinyl alcohol binder). The Li_4_Si_0.8_Ti_0.2_O_4_ ceramic pebbles sintered at 900 °C for 3 h in helium atmosphere. Figure 1 shows the complete preparation process of the Li_4_Si_0.8_Ti_0.2_O_4_ ceramic pebbles.

### 2.2. Method for Characterizing Pebbles Performance

The crystal structure of Li_4_Si_0.8_Ti_0.2_O_4_ ceramic pebbles identification was performed by X-ray diffractometry (BRUKER-D8 Advance, Bruker Corporation, Billerica, MA, USA). The grain size of the Li_4_Si_0.8_Ti_0.2_O_4_ ceramic pebbles were analyzed using the scanning electron microscope ((GeminiSEM 300, Carl Zeiss AG, Oberkochen, BW, Germany). Using helium as the medium, the density of the Li_4_Si_0.8_Ti_0.2_O_4_ ceramic pebbles were tested using an automatic true density tester (Ultrapyc 1200e, Anton Paar QuantaTec, Boynton Beach, FL, USA). The particle size distribution and sphericity of the ceramic pebbles were characterized using the dynamic image particle analysis system (BT-2900, Dandong Bettersize Instruments Co., Ltd., Dandong, Liaoning, China). The porosity of the pebbles were analyzed using the aperture analysis instrument (NOVA 2200e, Quantachrome Instruments Corp., Boynton Beach, FL, USA). At both room temperature and high temperature conditions, the crushing load of the Li_4_Si_0.8_Ti_0.2_O_4_ ceramic pebbles were measured using the universal material testing machine (AG-IC, Shimadzu Instruments (Suzhou) Co., Ltd., Suzhou, Jiangsu, China).

### 2.3. Tritium Release Experiments

The pebbles of Li_4_Si_0.8_Ti_0.2_O_4_ ceramic need to undergo neutron irradiation to obtain tritium. Before the neutron irradiation, the pebbles need to be pre-treated (the pebbles were dried for 2 h at 600 °C one week before irradiation) and placed in a quartz tube. Subsequently, these pebbles underwent 30 min of desorption heat treatment at 900 °C. During the heating process, the excess gas in the quartz tube was repeatedly replaced with pure helium. Finally, the pebbles were sealed in quartz capsules filled with helium. The weight of these pebbles was approximately 10 milligrams. Then, these quartz-sealed samples were exposed to a reference thermal neutron.

The neutron irradiation experiment was carried out in the experimental reactor (the China Mianyang Research Reactor, CMRR) of the Institute of Nuclear Physics and Chemistry (INPC), China Academy of Engineering Physics (CAEP). The Li_4_Si_0.8_Ti_0.2_O_4_ ceramic pebbles were subjected to two different thermal neutron irradiations, with neutron fluences of 1.15 × 10^19^ n cm^−2^ (irradiation time: 120 h) and 1.75 × 10^19^ n cm^−2^ (irradiation time: 240 h), respectively. The temperature of the Li_4_Si_0.8_Ti_0.2_O_4_ ceramic pebbles during neutron irradiation was approximately 60 °C. After the Li_4_Si_0.8_Ti_0.2_O_4_ ceramic pebbles have undergone neutron irradiation, they were cooled for six months and then removed from the CMRR. Subsequently, out-of-pile tritium release experiments were conducted; however, neutron spectrum-dependent parameters were not specifically evaluated, as experimental constraints prevented their determination.

**Figure 2:**
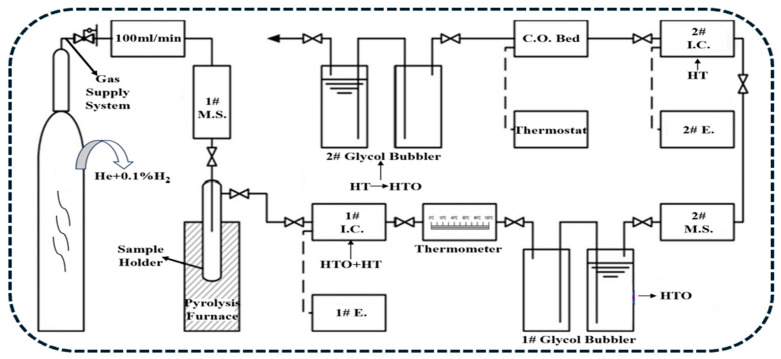
Out-of-pile tritium release system experimental device flow chart.

Experimental system: Tritium release experiments were performed using temperature-programmed thermal desorption in an out-of-pile tritium recovery system. The overall experimental setup is illustrated in Figure 2. The system mainly comprised two ionization chambers, two sets of glycol bubblers, a copper oxide bed, a pyrolysis furnace, a sample holder, and a gas supply system.

Tritium detection: The ionization chambers were used to measure the radioactive intensity of tritium carried away from the ceramic pebbles by the carrier gas, providing real-time tritium concentration data. Ionization chamber No. 1 was configured to detect total tritium, including both tritiated water (HTO) and tritium gas (HT). Ionization chamber No. 2 was dedicated to measuring tritium gas (HT) alone.

Tritium collection: The glycol bubblers served as the tritium collection system and played a critical role in the experimental procedure. The first set of glycol bubblers was used to directly collect tritiated water (HTO). The second glycol bubbler was used to collect oxidized tritium: the tritium gas in the carrier gas was passed through a copper oxide bed at approximately 400 °C, where it was oxidized into tritium water (HTO), thereby enabling efficient collection for subsequent analysis.

Experimental procedure: The irradiated sample, encapsulated in quartz, was placed in the sample holder. The quartz tube was then broken, and the sample was introduced into the pyrolysis furnace. The system was purged with a carrier gas composed of helium containing 0.1% hydrogen (He + 0.1% H_2_) until the ionization chamber signal dropped to a sufficiently low background level. Thereafter, the irradiated pebbles were heated from room temperature to 950 °C at a constant heating rate of 5 °C/min, followed by a holding period of 2 h at 950 °C. The entire experiment was conducted under a continuous flow of the same carrier gas at a flow rate of 100 mL/min.

Tritium quantification: After the completion of the tritium release experiment, the total tritium content was measured by means of a liquid scintillation counting (LSC) system. This method allowed for precise determination of tritium activity in the collected fractions, including both tritiated water and tritium gas.

## 3. Results and Discussions

### 3.1. Characterization of Li_4_Si_0.8_Ti_0.2_O_4_ Pebbles

**Figure 3:**
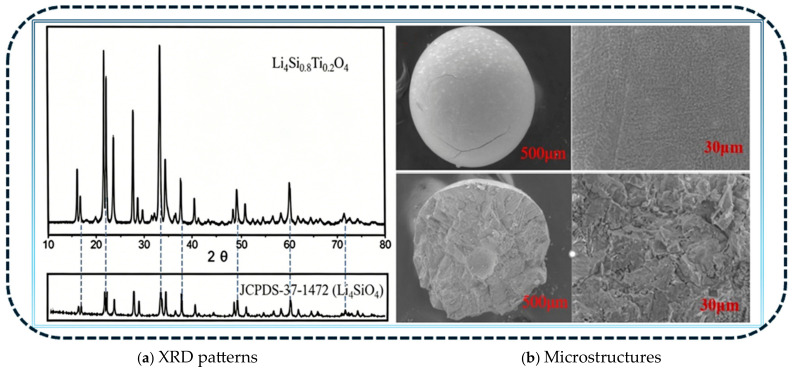
(**a**) XRD patterns of the Li_4_Si_0.8_Ti_0.2_O_4_ ceramic pebbles fired at 900 °C for 3 h. (**b**) Surface and cross-sectional microstructures.

The structure and microstructure of the prepared Li_4_Si_0.8_Ti_0.2_O_4_ ceramic balls prepared were characterized by XRD and SEM (Figure 3). As shown in Figure 3a, all the diffraction peaks of the ceramic pellets were highly consistent with the standard card of Li_4_SiO_4_ (JCPDS No. 37-1472), and no characteristic peaks of Li_2_TiO_3_ or TiO_2_ and other second phases were detected. Compared with pure Li_4_SiO_4_, the diffraction peaks of the sample were shifted to lower angles as a whole, which was consistent with the expected crystal cell expansion caused by the partial substitution of Ti^4+^ for Si^4+^ with a larger ionic radius. It should be noted that the conventional XRD data alone is not sufficient to strictly distinguish different structural models such as solid solutions, the coexistence of nanoscale second phases, and Ti substitution for Si positions. Therefore, further SEM observation was conducted to examine the microscopic structure of the sample. Figure 3b shows that the sample has a uniform structure at the micrometer scale, and no obvious segregation or abnormal aggregation was observed, which is the characteristic microstructure of the solid solution phase. More importantly, our team has conducted systematic research on the synthesis, phase composition, microscopic structure, and thermal physical properties of the Li_4_Si_1−x_Ti_x_O_4_ system (x = 0.1, 0.2, 0.3, 0.4) in the previous work [11]. Through evidence such as continuous peak shifts and the absence of impurity diffraction peaks, it was clearly confirmed that when x ≤ 0.3, Ti successfully occupied the Si position and formed a continuous solid solution. The composition of the sample studied in this paper is x = 0.2, and its XRD and SEM characteristics are basically consistent with those of the solid solution confirmed in the previous work. Therefore, based on the above evidence, the most accurate description of this material is Li_4_Si_0.8_Ti_0.2_O_4_ solid solution. Additionally, Figure 3b also indicates that the Li_4_Si_0.8_Ti_0.2_O_4_ ceramic pellets prepared by the wet method have excellent microscopic structure and excellent spherical morphology, and the sphericality is maintained during the sintering process. The sphericality of the obtained pellets is approximately 1.00. The upper half of Figure 3b shows the surface microscopic structure of the sintered ceramic pellets, and the lower half shows their cross-sectional morphology. From the microscopic structure diagram, it can be seen that the introduction of Ti atoms increases the grain size of the material matrix, with an average grain size of approximately 3–10 μm. The main characteristic parameters of Li_4_Si_0.8_Ti_0.2_O_4_ and Li_4_SiO_4_ pellets are summarized in Table 1. The term “porosity” mentioned in this article specifically refers to the open porosity, and its measured value is 7.9%. The specific measurement method is as follows: Firstly, use a pore size analyzer to measure the total pore volume of the ceramic ball sample, and combine with the apparent volume of the sample; directly calculate the open porosity using the following formula:

$$\gamma =\frac{V_{P}}{V_{P}+V_{S}}\times 100\%$$

where *V_P_* is the open pore volume and *V_S_* is the solid volume. At the same time, use a true density analyzer to measure the apparent density and true density of the sample, and obtain the total porosity (total porosity = 1 − apparent density/true density) by calculating the relative density. Then, subtract the open porosity from the total porosity to obtain the closed porosity. The above method clearly distinguishes the total porosity, open porosity, and closed porosity, and there is no confusion between open and closed pores. It should be noted that due to the influence of the measurement equipment’s accuracy and the measurement error of the sample volume, the measurement results have certain uncertainty. However, this result is consistent with the basic law between the density and porosity of ceramic materials (the lower the density, the greater the porosity) and therefore still has reference value.

### 3.2. Mechanical Properties of Li_4_Si_0.8_Ti_0.2_O_4_ Pebbles

**Figure 4:**
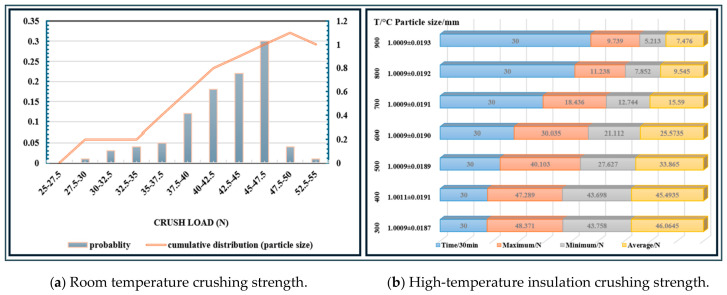
The compressive strength of Li_4_Si_0.8_Ti_0.2_O_4_ ceramic pebbles at room temperature and the compressive strength after being subjected to high temperature for 30 min. (**a**) The distribution of the compressive strength of the pebbles at room temperature. (**b**) The crushing strength of the pebbles after being heated and insulated at high temperature.

Excellent mechanical properties are crucial for solid tritium breeding pebbles. The mechanical properties of solid tritium breeding pebbles are usually characterized by the crushing load. For a systematic evaluation of the pebble mechanical behavior, measurements were conducted at room temperature and at elevated temperatures of 300 °C, 400 °C, 500 °C, 600 °C, 700 °C, 800 °C, and 900 °C (i.e., at 100 °C intervals). A constant dwell time of 30 min was applied for all high-temperature tests. For all the pebble samples treated at the corresponding temperature and duration, we conducted detailed crushing load tests one by one. Figure 4 shows the compressive strength of Li_4_Si_0.8_Ti_0.2_O_4_ ceramic pebbles at room temperature and the compressive strength after being subjected to high temperature for 30 min. A total of 100 Li_4_Si_0.8_Ti_0.2_O_4_ ceramic pebbles with particle sizes ranging from 0.2 to 1.1 mm were randomly selected for the testing of their room-temperature crushing performance. As the particle size increases, the statistical distribution of the crushing load shows a distinct single-peak and right-skewed characteristic, with the overall numerical range being 27.5–55 N. As shown in Figure 4a, in the relatively low-load range (0–25 N), the proportion was zero, indicating that all the pebbles had basic compressive resistance and no serious defects. The crush load was mainly concentrated in the range of 37.5 to 47.5 N, accounting for as high as 82% of the total. Among them, 37.5–40 N accounted for 12%, 40 to 42.5 N accounted for 18%, 42.5 to 45 N accounted for 22%, and 45 to 47.5 N accounted for 30%. The peak occurred at 45 to 47.5 N, indicating that the mechanical properties of most Li_4_Si_0.8_Ti_0.2_O_4_ ceramic pebbles were at a relatively high and concentrated level. The low-load zone (<35 N) accounted for only 13% of the total, covering a wide range of 25–35 N (27.5–30 N accounted for 1%, 30–32.5 N accounted for 3%, 32.5–35 N accounted for 4%, 35–37.5 N accounted for 5%). Although the overall proportion was not high, each interval had a distribution of 3–5%, indicating that low-load Li_4_Si_0.8_Ti_0.2_O_4_ ceramic pebbles were not extreme cases but that a certain proportion of the samples had their compressive strength reduced due to their particle size being too small or internal micro-defects. The high-load zone (>45 N) accounted for 5% (47.5–48 N accounted for 4%, 52.5–55 N accounted for 1%). This indicates that the Li_4_Si_0.8_Ti_0.2_O_4_ ceramic pebbles capable of achieving extremely high crushing loads are relatively scarce. This might be related to the upper limit of particle size, the extremely low porosity value, or the optimized range of grain size. There is a clear positive correlation between the load and the particle size: Figure 4a shows that for the high-load range, the Li_4_Si_0.8_Ti_0.2_O_4_ ceramic pebbles have a particle size of 0.6–1.0 mm (with the upper limit of the actual test being 1.1 mm), while in the low-load area, the pebbles mainly correspond to the small particle size group of 0.2–0.4 mm. This pattern may be related to the fracture mechanics behavior of ceramic materials. Under similar microstructural conditions, the absolute fracture load tends to increase with particle size. This can be attributed to the fact that during crushing tests, larger particles exhibit greater contact areas and volume effects, which generally require a higher external force to initiate fracture. However, an excessively broad particle size distribution (ranging from 0.2 to 1.1 mm) appears to have directly contributed to the dispersion of mechanical properties. Although the pebbles performed well under high-load conditions, the presence of a fraction (approximately 13%) that fractured under low loads could potentially reduce the overall mechanical reliability of the packed bed. In a long-term thermal cycling or irradiation environment, this subpopulation of weaker pebbles might serve as initiation sites for localized fragmentation, thereby compromising the long-term structural integrity of the bed.

Under long-term thermal cycling or irradiation conditions, the particle size may become the initiating factor for local fragmentation of the cladding tritium breeding pebble bed. Based on this and in combination with the results shown in Figure 4a, this study selected 100 ceramic balls with a diameter of approximately 1 mm, and their high-temperature crushing performance was tested under the same insulation time conditions. The ceramic balls used in this crushing strength test all came from samples prepared from the same batch. Before the test, all samples were placed in a tube furnace and dried at 200 °C under a high-purity argon atmosphere for 2 h to remove adsorbed moisture. Then, they were naturally cooled to room temperature and prepared for use. The high-temperature crushing test was conducted by testing a single ceramic ball successively. The specific steps were as follows: Place a single ceramic ball in the test furnace, heat it at a heating rate of 10 °C/min under a high-purity helium atmosphere to the target temperature, and hold it for 30 min to ensure uniform temperature distribution of the ball; then, maintain the target temperature constant, and conduct the crushing test at a loading rate of 0.5 mm/min, recording the maximum crushing load. After testing one sample, wait for the furnace temperature to cool to room temperature, then place the next new sample, and repeat the above heating, holding, and crushing steps until all 10 samples at this temperature point have been tested. When changing to a different temperature point, the equipment also needs to be cooled to room temperature before starting the test at the next temperature point. Based on the data presented in Figure 4b, it can be seen that at 300 °C and 400 °C, the Li_4_Si_0.8_Ti_0.2_O_4_ ceramic pebbles achieved relatively high compressive strength values. The maximum values reached above 37 N, the minimum values remained not lower than 33 N, and the average values were above 35 N. This level of mechanical performance may be considered indicative of adequate structural integrity for the purposes of this study. However, when the temperature rose to 500 °C, the strength dropped sharply, with the average value dropping to 33.865 N (maximum 40.103 N, minimum 27.627 N); thereafter, as the temperature increased, the strength continued to deteriorate—25.573 N at 600 °C, 15.590 N at 700 °C, 9.545 N at 800 °C, and less than 7.476 N at 900 °C. This observed pattern may suggest the existence of a critical temperature range for the pebbles, approximately between 350 °C and 400 °C. Once the temperature exceeds this range, the crushing performance appears to decline notably, and the temperature-induced loss in strength may exhibit an irreversible cumulative trend. Under thermal cycling conditions, the degradation of compressive strength caused by exposure to elevated temperatures could indeed increase the risk of certain pebbles becoming local sources of fragmentation within the packed bed. That said, this finding does not necessarily imply that operation at temperatures above 400 °C should be entirely ruled out. Instead, a comprehensive assessment of specific operating conditions, safety environment, and acceptable fragmentation probability is required. According to the data, the strength is excellent in the range of 300 °C to 400 °C (average value > 45 N). At 500 °C, the average value drops to 33.865 N, a reduction of approximately 25%. At 600 °C, it further drops to 25.573 N, and above 700 °C, it is less than 15.590 N. This indicates that 400 °C is a significant performance inflection point. If the operating temperature remains above this value for a long time, more stringent quality inspections or shorter replacement cycles are required.

Based on the above considerations, the research team further examined the potential cumulative effect of the irradiation environment on the performance of the Li_4_Si_0.8_Ti_0.2_O_4_ ceramic pebbles. It is possible that neutron or gamma irradiation may induce lattice defects, grain boundary embrittlement, or micro-crack propagation, which could lead to a further reduction in crushing strength at a given temperature and might even cause a shift in the inflection point temperature toward lower values. A comparison of the crushing performance of Li_4_SiO_4_ and Li_4_Si_0.8_Ti_0.2_O_4_ ceramic pebbles prepared by different methods is presented in Table 2. For Li_4_SiO_4_ ceramic pebbles, the differences in crush load were systematically compared across various preparation methods, including the wet process, sol-gel method, hydrothermal method, extrusion–spheronisation method, and melt spraying method. Each of these methods, owing to its distinct granulation mechanism, tends to produce pebbles with different microstructural characteristics, such as variations in density, grain size, porosity, and defect distribution, all of which may significantly influence the resulting mechanical properties. For instance, pebbles prepared by the wet method generally exhibit good plasticity and uniformity; however, sintering often leaves behind micropores, leading to moderate compressive strength. The sol-gel method tends to form a denser gel network, and after sintering, the resulting pebbles typically have fine grains and low porosity, thereby achieving relatively high crush loads. The hydrothermal method allows for the synthesis of high-crystallinity powders at comparatively low temperatures; the resulting pebbles tend to have fewer defects and exhibit excellent strength. The extrusion–spheronisation method is straightforward and offers high yield, but the internal structure of the pebbles is prone to develop layered or strip-like features, which may affect mechanical properties, often resulting in crush loads slightly lower than those achieved by the sol-gel or hydrothermal methods. The melt spraying method produces dense, pore-free pebbles through rapid solidification, yielding relatively uniform compressive strength; nevertheless, its applicability may be limited to specific material systems.

Furthermore, a detailed comparison of the crush load was made between Li_4_Si_0.8_Ti_0.2_O_4_ ceramic pebbles prepared by the hydrothermal method and those prepared by the wet method. The results suggest that both methods produce pebbles with comparable compressive strength, and importantly, both are significantly higher than those observed for control samples without the Ti addition. This may indicate that the introduction of Ti atoms could effectively enhance the intrinsic mechanical properties of the ceramic pebbles, leading to a notable improvement in their compressive crush load. For the Li_4_Si_0.8_Ti_0.2_O_4_ ceramic pebbles prepared by the wet method, crush load data after irradiation were also obtained, providing a basis for further evaluation of irradiation-induced mechanical degradation. It should be noted that due to the limited number of samples after irradiation, the crushing strength data obtained in this study is relatively limited. Compared with the test results under normal temperature conditions before irradiation, the crushing strength of the irradiated samples has decreased, suggesting that irradiation may cause a certain degree of softening or brittleness. However, none of the test spheres completely broke, and their structural integrity was still largely maintained.

### 3.3. Tritium Release Behavior of the Irradiated Li_4_Si_0.8_Ti_0.2_O_4_ Ceramic Pebbles

It should be noted that the irradiation conditions adopted in this work differ significantly from those expected in a fusion blanket. The Li_4_Si_0.8_Ti_0.2_O_4_ ceramic pebbles were sealed in quartz capsules and irradiated at a temperature of approximately 60 °C, whereas the typical operating temperature range for a solid-state tritium breeder blanket is 400–900 °C. This temperature difference has significant effects on the irradiation damage behavior; defect recombination kinetics; and the generation, migration and aggregation of transmutation gases (especially helium and tritium). These understandings are also of reference value for analyzing similar phenomena in the fusion environment. With the gradual establishment and improvement of domestic fusion neutron source facilities, it will become feasible in the future to irradiate tritium breeder materials with genuine fusion neutrons, thereby obtaining experimental data that are more representative of fusion reactor conditions.

Owing to the high radioactivity of the as-irradiated samples, the following objective constraints exist regarding experimental conditions. First, the handling and transfer of the samples must be carried out in a hot cell or under shielded conditions, which makes it difficult to directly perform conventional microstructural characterizations. Second, to avoid additional interference or contamination of the samples and to ensure the safety of the experimental personnel, this study did not conduct any other characterizations of the irradiated pebbles that involve complex sample preparation or prolonged operations. Therefore, the focus of this paper is placed on the offline tritium release behavior of the samples after fission neutron irradiation, namely, measuring the tritium release temperature and release form via temperature-programmed thermal desorption experiments. This experimental method relies on existing laboratory facilities and can directly reflect the tritium release characteristics of breeder materials after neutron irradiation. This study focuses on understanding the generation and retention of tritium as well as the interaction between materials and tritium from a qualitative perspective. It is hoped that this will provide some reference for the design of tritium plants and related tritium safety work.

**Figure 5:**
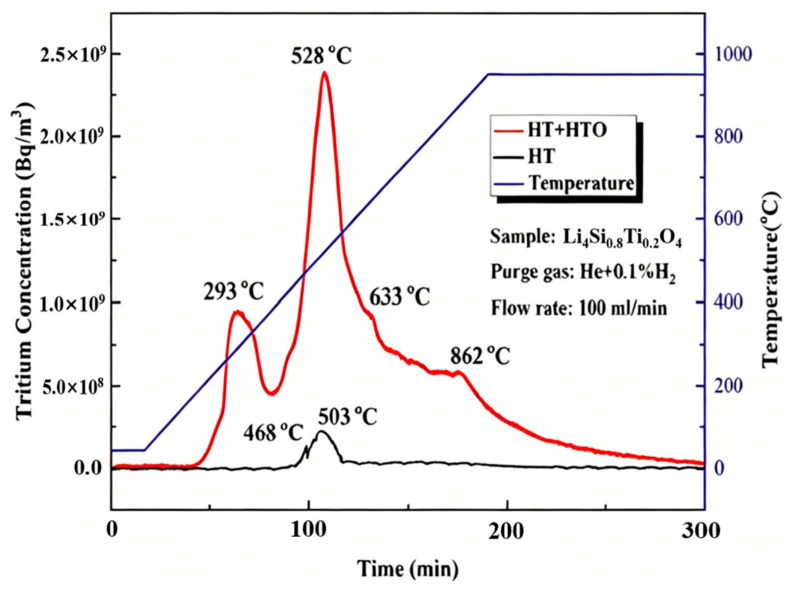
The tritium release curve of Li_4_Si_0.8_Ti_0.2_O_4_ ceramic pebbles during the experiment after the first irradiation.

Figure 5 shows the tritium release curve of Li_4_Si_0.8_Ti_0.2_O_4_ ceramic pebbles during the experiment after the first irradiation. This graph shows the tritium release data of the Li_4_Si_0.8_Ti_0.2_O_4_ ceramic pebbles after it was irradiated by the first neutron (1.15 × 10^19^ n cm^−2^, 120 h). In the figure, “HT” denotes tritium gas, while “HTO” represents tritiated water (tritium water). The red curve represents the total tritium release curve, while the black curve shows the tritium release. The total release curve displays three pronounced peaks along with one minor peak. The four peaks are located at 293 °C, 528 °C, 633 °C, and 862 °C, respectively. The tritium gas curve shows two distinct peaks at 468 °C and 56 °C. A comparison between the total tritium release curve and the tritium gas curve indicates that tritium was released primarily in the form of tritium gas. The liquid scintillation analysis results show that the proportion of tritium water release was 89.1%, while the proportion of tritium gas was only 10.9%. The radioactive tritium water begins to release at 180 °C, and a smaller release peak appears around 293 °C. This observation may be attributed to the presence of 0.1% hydrogen in the carrier gas, which likely facilitates an isotope exchange reaction between hydrogen and tritium, thereby leading to the formation and subsequent release of tritium water. The presence of 0.1% hydrogen in the carrier gas is likely responsible for this phenomenon. This hydrogen can promote an isotope exchange reaction with tritium, converting tritium gas into tritiated water. As a result, tritium is released primarily in the form of tritium water.

The carrier gas was not pure helium due to the possibility that radiation-induced damage to the sample might have compromised the acquisition of a reliable tritium release curve under such conditions. A weak tritium release peak was observed at 633 °C, which might be related to the thermal decomposition of the [=Ti–T] groups in the Li_4_Si_0.8_Ti_0.2_O_4_ ceramic pebbles. The temperature at which the Li_4_Si_0.8_Ti_0.2_O_4_ ceramic pebbles released tritium did not fall into a lower temperature range, indicating that the samples did not suffer any damage during irradiation and cooling, and the packaging remained intact. The large amount of tritium release might be due to the water formed by the hydrogen–oxygen flame during sample sealing adhering to the surface of the samples. Specifically, during the sealing process with hydrogen–oxygen flame, the sample may generate water vapor through combustion reactions and then adsorb CO_2_ and H_2_O in the cooling and subsequent storage and operation processes, thereby forming hydroxyl, carbonate, or bicarbonate species on the sample surface. During the temperature-programmed desorption (TPD) process, these surface species can desorb or decompose at lower temperatures (such as 200–350 °C), releasing H_2_O, CO_2_, and possibly surface-adsorbed or near-surface tritium in the form of HTO or HT through isotope exchange or carrying action, thus forming a seemingly “low-temperature release” peak. It is worth noting that this release induced by surface contaminants is not an inherent tritium release behavior of the material (such as Ti-T bond breakage or isotope exchange in the lattice) but may overlap with or dominate the low-temperature peak observed at 293 °C. A comparison of the tritium release temperatures for Li_4_SiO_4_, Li_4.2_Si_0.8_Al_0.2_O_4_, and Li_4_Si_0.8_Ti_0.2_O_4_ ceramic pebbles is summarized in Table 3. Currently, there are no conditions available to conduct blank unirradiated sphere desorption experiments, which is a limitation of this work. Further research is needed when conditions are mature in the future. According to the LSC test results, the total amount of tritium released during the experiment was approximately 7.85 mCi. The results show that the released tritium is predominantly in the form of HTO, which is beneficial for tritium recovery and long-term storage. The calculated total recovery rate is approximately 92%. The detailed data are listed in Table 4.

**Figure 6:**
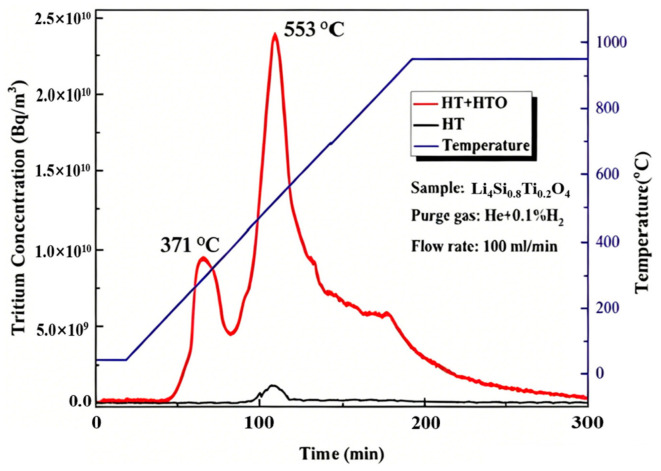
The tritium release curve of Li_4_Si_0.8_Ti_0.2_O_4_ ceramic pebbles during the experiment after the second irradiation.

The second batch involved higher irradiation doses and longer irradiation times. As a result, the ionization chamber continued to exhibit poor stability throughout the off-line tritium release experiment. This persistent instability introduced considerable challenges, making it far more difficult to conduct the experiment reliably and obtain consistent measurements. After the second irradiation (1.75 × 10^19^ n cm^−2^, 240 h), the tritium release behavior of the Li_4_Si_0.8_Ti_0.2_O_4_ ceramic pebbles was measured, and the resulting release curve is shown in Figure 6. As the irradiation dose increased and the irradiation time was extended, the tritium release temperature of the Li_4_Si_0.8_Ti_0.2_O_4_ ceramic pebbles showed a modest increase relative to that observed after the first irradiation. This shift toward higher release temperatures implies that under more severe irradiation conditions, the release of tritium required more thermal energy, suggesting an alteration in the tritium retention or diffusion characteristics within the ceramic pebbles. This result also points to a discernible trend: a higher neutron fluence rate leads to a progressive shift in the tritium release peak toward higher temperatures for the irradiated ceramic pebbles. Such a shift implies that under increased irradiation intensity, tritium becomes more tightly retained within the pebbles, and its effective release can only occur when sufficiently high thermal energy is supplied. Results from the off-line tritium release experiment under elevated irradiation dose conditions showed that the tritium release peak of the Li_4_Si_0.8_Ti_0.2_O_4_ ceramic pebbles shifted toward the high-temperature region; in other words, the tritium release temperature increased. In contrast, the overall shape and peak characteristics of the tritium release curve did not undergo significant changes. This phenomenon might reflect an increase in tritium generation caused by the higher irradiation dose. As a possible consequence, the desorption process, which is controlled by bulk diffusion, may necessitate more stringent thermal activation conditions to take place, thereby resulting in a rise of the desorption peak temperature.

Compared to the first irradiation, the tritium release peaks of the Li_4_Si_0.8_Ti_0.2_O_4_ ceramic pebbles observed in the second irradiation appeared somewhat simpler and more clearly defined, although the substantial changes remained limited. The relative proportions of tritium water and tritium gas showed little variation, with the primary differences being a moderate increase in tritium release temperature and a sharper peak shape. Overall, the relative ratio between the two chemical forms did not differ significantly from that observed in the first batch. It is worth noting that all irradiated pebbles were prepared using a wet chemical method, which resulted in relatively large grain sizes. This microstructural characteristic may have two main consequences. First, the diffusion path length for tritium within the grains is effectively extended. Second, the presence of larger grains tends to reduce the specific surface area as well as the density of surface desorption activation sites, which in turn may slow down the surface desorption rate of tritium. Additionally, the observed increase in tritium release temperature appears to be accompanied by a gradual rise in the proportion of tritium released as tritium gas.

Through two batches of irradiation experiments, it was found that a relatively large fraction of tritium was released in the form of tritium water from the Li_4_Si_0_._8_Ti_0_._2_O_4_ ceramic pebbles after irradiation. In addition, a gradually increasing trend in tritium release temperature was observed as irradiation time and irradiation dose were extended. This suggests that prolonged and more intense irradiation may impose higher thermal requirements for effective tritium release. This change is particularly significant in the main peak region around 500 °C. This peak is primarily attributed to the desorption process controlled by the bulk diffusion of tritium species (such as T_2_ or HT) through the ceramic pebble matrix. However, other contributing factors cannot be ruled out. For instance, the presence of surface trap sites or grain boundaries may temporarily retain tritium, requiring additional thermal energy for release. Additionally, the isotope exchange reaction between tritium and residual hydrogen or hydroxyl groups on the material surface could also play a role, leading to the formation of tritiated water (HTO) and shifting part of the release to higher temperatures. Furthermore, radiation-induced defects within the crystal lattice—which tend to accumulate under higher irradiation doses—may act as trapping sites for tritium, thereby delaying its release and contributing to the prominence of this peak. Therefore, the observed peak around 500 °C likely reflects a combination of diffusion-controlled desorption, defect-mediated trapping, and surface exchange processes. The release mechanism is primarily governed by the surface desorption process, which is speculated to be closely associated with the thermal decomposition of the Li-T group [6,8]. Overall, the Ti-doped Li_4_Si_0.8_Ti_0.2_O_4_ samples exhibited a notably lower tritium release temperature compared to both the undoped Li_4_SiO_4_ breeder pebbles and the Al-doped Li_4_SiO_4_ counterparts. This suggests that the incorporation of titanium may facilitate the desorption process by modifying the local chemical environment or reducing the thermal stability of Li-T bonds. This result may imply that Ti doping could alter the microstructure or surface chemical state of the material, thereby potentially reducing the energy barrier for tritium release. One possible explanation is that the incorporation of Ti atoms effectively lowers the desorption activation energy at the sample surface, which in turn may significantly enhance the migration and diffusion rate of tritium within the grains.

Under high neutron flux irradiation conditions, various types of defects are likely to be induced within the Li_4_Si_0.8_Ti_0.2_O_4_ ceramic pebbles. These defects are presumably associated with tritium molecules, and it is further speculated that they may originate primarily from the formation of [Ti–T] and [Si–T] groups. It is worth noting that owing to the prolonged irradiation time and high thermal neutron fluence, both batches of irradiated samples accumulated a considerable number of irradiation-induced defects. These defects appear to exhibit a strong trapping capability for tritium, and their quantity and type may serve as important factors influencing the overall tritium release behavior. The deuterium release peaks observed in both groups of graphs are tentatively attributed to the thermal decomposition of high-temperature stable [≡Si−T] groups. These groups are thought to be formed through the reaction between [≡Si^0^] and [T^0^] under high neutron flux irradiation, and they tend to decompose only at elevated temperatures, thereby giving rise to the corresponding desorption peaks. The high-temperature release behavior of tritium could pose certain challenges to the compatibility of adjacent materials within the breeder blanket. Nevertheless, the newly developed Li_4_Si_0.8_Ti_0.2_O_4_ ceramic pebbles have demonstrated a relatively lower tritium release temperature, which may be of positive significance for the future development and design of breeder blankets. Table 5 integrates the offline tritium release data after two neutron exposure doses. These data correspond to Figure 5 (for the first irradiation) and Figure 6 (for the second irradiation).

## 4. Conclusions

In summary, Li_4_Si_0.8_Ti_0.2_O_4_ ceramic pebbles were successfully prepared by a wet method using the powders synthesized by solid-state reaction. Compared with the Li_4_SiO_4_ ceramic pebbles, the Li_4_Si_0.8_Ti_0.2_O_4_ ceramic pebbles appear to retain the original advantages and, under the present test conditions, exhibit certain differences in mechanical properties at both room temperature and high temperatures. Whether these differences imply any overall performance advantage requires further investigation. In this study, an offline tritium release experiment using the programmed temperature desorption method was conducted. The tritium release behavior of Li_4_Si_0.8_Ti_0.2_O_4_ ceramic pebbles during heating was characterized as part of an effort to assess their release performance. When compared with Li_4_SiO_4_ ceramic pebbles, the Ti-doped pebbles showed a tendency toward tritium release at relatively lower temperatures. Specifically, the tritium release temperature corresponding to the main peak gradually decreased, with the most pronounced decrease observed for the first batch of irradiated pebbles. One possible interpretation—though remaining to be confirmed—is that the increase in Ti doping may have led to the formation of desorption sites with lower activation energy on the sample surface, which could facilitate the migration and diffusion of tritium within the grains.

## Figures and Tables

**Table 1:** Characteristics of the Li_4_Si_0.8_Ti_0.2_O_4_ and Li_4_SiO_4_ pebbles.

Characteristics	Li_4_SiO_4_ Pebbles [6]	Li_4_Si_0.8_Ti_0.2_O_4_ Pebbles
Grain size	3.5 µm	3–10 µm
Sphericity	≤1.02 (a.v.)	1.00 ± 0.015 (a.v.)
Particle size	0.5–1.0 mm	0.5–1.10
Li enrichment	7.5%	7.5%
Open porosity		7.9%
density	80% TD	80.9% TD

**Table 2:** The comparison of crushing performance of Li_4_SiO_4_ and Li_4_Si_0.8_Ti_0.2_O_4_ ceramic pebbles under different methods was presented.

Ceramic Pebbles	Preparation Method	Minium (N)	Maximum (N)	Average (N)	Irradiated (N)
Li_4_SiO_4_	Wet process	4.9406	35.1000	17.1315	
	Sol-gel	5.2438	41.0438	24.3620	/
	Hydrothermal	7.1156	34.1063	21.2158	/
	Spheronisation	8.0156	100.6810	42.9874	/
	Molten-droplet	8.2000	35.2313	17.9366	/
	Melt spraying	6.3563	37.0719	21.6259	/
Li_4_Si_0.8_Ti_0.2_O_4_	Hydrothermal [25]	/	/	52	/
	* Wet process	43.758	48.371	46.0645	7.391/5.312

* In this work.

**Table 3:** This table compares the tritium release performance of Li_4_SiO_4_, Li_4.2_Si_0.8_Al_0.2_O_4_, and Li_4_Si_0.8_Ti_0.2_O_4_ ceramic pebbles.

Ceramic	Preparation Method	Samples	Irradiation Dose	Purge Gas Condition	T. of Tritium Release (°C)
Li_4_SiO_4_ [8]	Wet process	pebbles	4.26 × 10^16^	He + 0.1% H	477
			2.772 × 10^17^	He + 0.1% H	485
Li_4.2_Si_0.8_Al_0.2_O_4_ [8]	Solid-phase reaction	powders	4.0194 × 10^18^	He	548
Li_4_Si_0.8_Ti_0.2_O_4_	* Wet process	pebbles	1.15 × 10^19^	He + 0.1% H	293
			1.75 × 10^19^		371

* In this work.

**Table 4:** The main results of tritium release.

Ceramic	Samples	Irradiation Dose	HT + HTO (mCi)	HTO (mCi)	Ratio of HTO
Li_4.2_Si_0.8_Al_0.2_O_4_ [8]	powders	4.0194 × 10^18^	5.6	4.57	82%
Li_4_SiO_4_ [8]			5.46	4.49	
* Li_4_Si_0.8_Ti_0.2_O_4_	pebbles	1.15 × 10^19^	7.85	7.22	89.1%
		1.75 × 10^19^	8.91	8.15	

* In this work.

**Table 5:** Summary of off-line tritium release data after two neutron irradiation doses, corresponding to Figure 5 (first irradiation) and Figure 6 (second irradiation).

Time (min)	HT + HTO				HT			
	Temperature (°C)		Tritium Concentration (Bq/m^3^)		Temperature (°C)		Tritium Concentration (Bq/m^3^)	
	Figure 5	Figure 6	Figure 5	Figure 6	Figure 5	Figure 6	Figure 5	Figure 6
50	200	200	0	0	200	200	0	0
60	300 (**293**)	300	1.0 × 10^9^	7.5 × 10^9^	300	300	0	0
80	400	400 (**371**)	1.0 × 10^9^	1.0 × 10^10^	400 (**468**)	400	1.5 × 10^8^	1.5 × 10^9^
100	500 (**528**)	500	1.75 × 10^9^	2.25 × 10^10^	500 (**503**)	500 (**510**)	1.5 × 10^8^	1.5 × 10^9^
110	550	550 (**553**)	1.75 × 10^9^	2.25 × 10^10^	550	550 (**535**)	1.5 × 10^8^	1.5 × 10^9^
140	600 (**633**)	600 (**650**)	1.50 × 10^9^	1.40 × 10^10^	600	600	1.75 × 10^8^	1.75 × 10^9^
170	850 (**862**)	850	5.0 × 10^8^	6.0 × 10^9^	850	850	0	0
200	950	950	2.5 × 10^8^	2.5 × 10^9^	950	950	0	0
300	950	950	0	0	950	950	0	0

## Data Availability

The original contributions presented in this study are included in the article. Further inquiries can be directed to the corresponding authors.
